# The use of biostimulants as a key to sustainable hydroponic lettuce farming under saline water stress

**DOI:** 10.1186/s12870-024-05520-8

**Published:** 2024-08-28

**Authors:** Boran İkiz, Hayriye Yildiz Dasgan, Sibel Balik, Sebnem Kusvuran, Nazim S. Gruda

**Affiliations:** 1 Department of Horticulture, Faculty of Agriculture, University of Cukurova, Adana, 01330 Türkiye; 2 Food and Agriculture Vocational School, Cankiri Karatekin University, Çankırı, 18100 Türkiye; 3 Institute of Plant Sciences and Resource Conservation, Division of Horticultural Sciences, University of Bonn, Bonn, Germany

**Keywords:** *Lactuca sativa* L. var*. crispa*, NaCl stress, Coping with stress, Soilless culture, Yield, Quality

## Abstract

**Backround:**

The utilization of high-quality water in agriculture is increasingly constrained by climate change, affecting availability, quality, and distribution due to altered precipitation patterns, increased evaporation, extreme weather events, and rising salinity levels. Salinity significantly challenges salt-sensitive vegetables like lettuce, particularly in a greenhouse. Hydroponics water quality ensures nutrient solution stability, enhances nutrient uptake, prevents contamination, regulates pH and electrical conductivity, and maintains system components. This study aimed to mitigate salt-induced damage in lettuce grown via the floating culture method under 50 mM NaCl salinity by applying biostimulants.

**Results:**

We examined lettuce’s physiological, biochemical, and agronomical responses to salt stress after applying biostimulants such as amino acids, arbuscular mycorrhizal fungi, plant growth-promoting rhizobacteria (PGPR), fulvic acid, and chitosan. The experiment was conducted in a greenhouse with a randomized complete block design, and each treatment was replicated four times. Biostimulant applications alleviated salt’s detrimental effects on plant weight, height, leaf number, and leaf area. Yield increases under 50 mM NaCl were 75%, 51%, 31%, 34%, and 33% using vermicompost, PGPR, fulvic acid, amino acid, and chitosan, respectively. Biostimulants improved stomatal conductance (58–189%), chlorophyll content (4–10%), nutrient uptake (15–109%), and water status (9–107%). They also reduced MDA content by 26–42%. PGPR (1.0 ml L^‒1^), vermicompost (2 ml L^‒1^), and fulvic acid (40 mg L^‒1^) were particularly effective, enhancing growth, yield, phenol, and mineral content while reducing nitrate levels under saline conditions.

**Conclusions:**

Biostimulants activated antioxidative defense systems, offering a sustainable, cost-effective solution for mitigating salt stress in hydroponic lettuce cultivation.

## Introduction

The current trajectory of global population growth is expected to continue throughout this century. Projections suggest that the world population will reach 9.7 billion by 2050 and 10.9 billion by 2100 [1]. The coexistence of malnutrition, over nutrition, and environmental pollution stemming from food-related activities is widespread globally. These intertwined issues share common determinants and necessitate comprehensive and integrated solutions. Concurrently, climate change emerges as a primary driver of risks, jeopardizing food security, plant ecosystems, and the nutritional quality of diets [1]. Climate, defined as the long-term variations in atmospheric conditions within a particular region, significantly influences agricultural practices [2]. Climate change impacts a wide range of stakeholders, including consumers, producers, media, and suppliers, on a global scale. These deviations from average atmospheric variables have increasingly exacerbated the frequency and intensity of various stressors on agricultural crop yield and quality [3, 4]. Agriculture is very vulnerable to climate change and is the most affected sector. Bisbis et al. [5] demonstrated that this also applies to the production of greenhouse vegetables. Exposing crops to abiotic stresses such as drought and salinity triggered by climate change negatively affects sustainable food production [6]. Agriculture, constituting approximately one-third of the Earth’s terrestrial surface, occupies a substantial portion of the global land area. The agricultural sector significantly influences anthropogenic water consumption, accounting for 70% of water utilization [7]. The prominence of agriculture in this context underscores its pivotal role in shaping globaldynamics. It emphasizes the imperative for informed water resource management strategies within the agricultural sector to foster sustainability and mitigate potential ecological ramifications. Suboptimal water resource management practices within the watershed pose significant challenges. This is evident in the excessive water use for irrigation, leading to substantial wastage of water resources [8]. Furthermore, unchecked pollution of water bodies exacerbates the problem, further threatening the sustainability of water reservoirs. Additionally, a noticeable decline in groundwater levels adds to the agricultural sector’s multifaceted challenges [9].

Salinity in soil and water is a widespread challenge, significantly inhibiting global agricultural food production, particularly in arid and semi-arid regions. The incidence of salt stress has become a widespread problem affecting approximately 20% of the world’s arable land, with an expectation that it will increase to 50% by the end of the 21st century [10]. The scarcity of water resources often obligates using saline groundwater for irrigation purposes in agricultural activities [11]. Elevated salinity levels induce damage at the molecular level to DNA, RNA, proteins, and lipids. At the cellular level, salt stress triggers osmotic and ionic stress, disrupts gas and nutrient exchange, and leads to the overproduction of reactive oxygen species (ROS) molecules such as hydrogen peroxide (H_2_O_2_), hydroxyl radical (OH^•^), superoxide (O2^•‒^), and singlet oxygen (^1^O_2_) [12, 13]. ROS diminishes cell expansion and metabolic activity, induces stomatal closure, alters photosynthesis, and disrupts carbon fixation, reducing growth, development, photosynthesis, and yield [14, 15]. Moreover, there’s an increasing strategy of using drainage and wastewater for irrigation, reflecting a growing necessity to maximize available water resources in the face of escalating water scarcity issues [16]. Therefore, there is an increasing need to enhance awareness regarding saline water management [11, 17].

Soilless culture is an agricultural technology that uses water sparingly in this context. It represents a contemporary method of plant cultivation in which plants are grown using inert organic or inorganic substrates. Typically, this cultivation technique involves using nutrient solutions in combination with the substrates above to provide plants with the necessary nutrients [18]. Soilless culture systems generally allow flexibility and intensification, ensuring high crop yields and high-quality products even in regions with unfavorable growing conditions [19]. These systems provide efficient tools to manage saline stress on plants, preventing salinity levels higher than the tolerance threshold of crops, which negatively affect plant growth and yield. Concurrently, controlled saline stress can be applied to increase secondary metabolites (phytochemicals/antioxidants) and sensorial quality traits (color, firmness, aroma) [20, 21] and to reduce anti-nutritional factors such as nitrate [22], improving the “whole” quality of vegetable products.

Biostimulants represent innovative agronomic tools positioned between fertilizers and plant growth regulators, demonstrating a unique ability to enhance plant growth and productivity. Biostimulants strengthen the efficacy of nutrient utilization within plants, bolster resilience against adverse environmental factors, elevate the caliber of produce, and facilitate optimal uptake of nutrients in scant quantities within the soil and root system [23, 24]. According to the most recent European regulation on fertilizers, these substances are classified based on their concentrations and consist of organic or inorganic products containing bioactive compounds and/or microorganisms. Applying biostimulants to the plant or rhizosphere improves nutrient absorption and assimilation efficiency, augments tolerance to abiotic stresses, and enhances the overall quality of the agricultural product. These effects are observed independently of the nutrient content of the biostimulants. This regulatory definition encapsulates the multifaceted role of biostimulants in modern agriculture [25, 26].

The Plant Growth-Promoting Rhizobacteria (PGPR) play a crucial role in mitigating salinity stress by enhancing water absorption capabilities, facilitating the uptake of essential nutrients, and accumulating osmolytes such as proline, glutamate, glycine betaine, soluble sugars, and carbohydrates [27, 28]. Additionally, PGPR contributes to the augmentation of antioxidative enzymes [29–31]. Vermicompost promotes biodiversity by fostering beneficial microorganisms, thereby enhancing plant growth by directly synthesizing plant growth-regulating hormones and enzymes. Furthermore, it indirectly aids in plant development by mitigating the impact of plant pathogens, nematodes, and other pests, thus strengthening plant health and reducing yield losses [32]. Arbuscular Mycorrhizal Fungi (AMF) serve as crucial root symbionts, playing a pivotal role in enhancing the growth of crop plants and helping host plants acquire tolerance to abiotic stressors such as salinity and drought [33–36]. Fulvic acid, hypothesized to originate from microbial metabolic processes, acts as a stimulatory agent in protecting crops from the adverse effects of salt stress [37, 38]. Chitosan is a natural biopolymer derived from chitin, found in the exoskeletons of crustaceans such as shrimp, crab, and lobster. It is produced by deacetylation of chitin, resulting in a positively charged polysaccharide [39, 40]. Chitosan, due to its biocompatibility, biodegradability, non-toxicity, and antimicrobial properties, is used in agriculture as a biopesticide, biofertilizer, and biostimulant to enhance plant growth, improve crop yield, and protect against abiotic stresses and diseases [41, 42].

Lettuce (*Lactuca sativa* L.), a prominent leafy vegetable in the *Asteraceae* family, holds significant nutritional value. It is a rich source of essential vitamins, including A, C, folate, and K, contributing to immune system support and optimal bone health maintenance. The presence of antioxidants, such as flavonoids, phenolic acids, and carotenoids, plays a crucial role in protecting the body against the harmful effects of free radicals, thereby reducing the risk of cellular damage and associated diseases [43]. It is commonly consumed as a fresh salad or minimally processed food, such as fresh-cut and mixed salads. Its consistent year-round consumer demand highlights its enduring popularity in the market [44]. Lettuce is known for its ease of cultivation and short growth cycleand is well-suited for year-round hydroponic cultivation. As a leading leafy vegetable, lettuce is a primary choice for hydroponic cultivation [45]. Moreover, lettuce is a sensitive vegetable to salt stress. This can significantly reduce yield and crop quality [45–47].

This study aims to assess the impact of sustainable and environmentally friendly biostimulant practices on enhancing the yield and quality of hydroponically grown lettuce using saline irrigation water. Lettuce, the foremost leafy vegetable, has been chosen for this study. Efficiently utilizing saline waters is paramount, particularly in regions facing water salinity challenges. Greenhouse hydroponic systems offer a sustainable solution for cultivating crops like lettuce, where water quality plays a pivotal role. Maintaining high water quality ensures optimal nutrient delivery and uptake efficiency, which is essential for maximizing yields and minimizing environmental impact. As such, integrating technologies that promote water conservation and saline water management in hydroponic setups enhances agricultural productivity and contributes to sustainable water resource management. Biostimulants may play a crucial role in improving efficiency whenusing saline waters. We hypothesize that applying amino acids, AMF, PGPR, vermicompost, fulvic acid, or chitosan will mitigate salt stress. Additionally, we anticipate observing alterations in the activity of antioxidant enzymes, changes in antioxidant levels, and mineral content in lettuce leaves, indicative of the effects of salt stress mitigation mediated by biostimulants. This study is among the first to comprehensively compare and examine six biostimulants in greenhouse hydroponic lettuce cultivation under 50 mM saline conditions.

## Materials and methods

### Experimental design and growth conditions

The study was carried out in a glasshouse 36°59′N, 35°18′E, at an elevation of 20 m above sea level during the autumn and winter seasons of 2021–2022 in a Mediterranean climate. During the daytime, temperatures inside the greenhouse fluctuated between 20 and 24 °C, while at night, they ranged from 13 to 16 °C. The relative humidity remained at 60–70%, and the plants received exposure to natural sunlight.

**Fig. 1:**
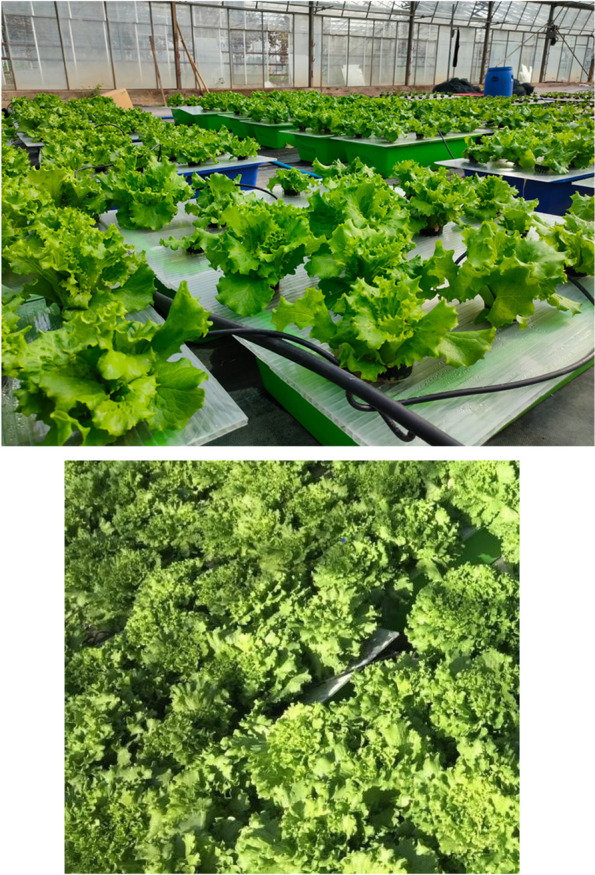
The experiment layout in the greenhouse involved growing lettuce in a floating culture system with biostimulants under 50 mM saline water. Application of biostimulants into the root medium

The Batavia type of lettuce (*Lactuca sativa* L. var. *crispa*), specifically the ‘Caipira’® cultivar from Enza Zaden seed company, was used as plant material. A hydroponic system using 50-L cultivation containers was set up, with plant roots submerged in aerated nutrient solution. The experiment followed a randomized complete block design with four replicates per treatment and ten plants per replicate, each tank serving as one replicate (Fig. 1). The distance between the rows of lettuce plants was 15 × 15 cm, with a plant density of 44.44 plant m^−2^. The lettuce plants were grown with the following nutrient solution in control treatment [48] (in mg L^‒1^): N (200), P (50), K (300), Ca (200), Mg (65), Fe (5.0), Mn (0.8), Cu (0.3), Zn (0.3), B (0.3), and Mo (0.05). The hydroponically grown lettuce plants were treated with 50 mM NaCl salinity. The biostimulants of AMF, amino acids, fulvic acid, chitosan, PGPR, and vermicompost were applied to a 50 mM NaCl salinized nutrient solution. Lettuce plants were grown in a floating culture system for 45 days and harvested. The pH of the nutrient solution was diligently maintained within the range of 6.0–6.2, while the electrical conductivity (EC) values were incrementally elevated to 1.3, 1.8, and 2.0 dS m^‒1^ levels in the control application throughout plant growth.

### Biostimulant applications

Amino acid and fulvic acid, products of “Köklü Group” company, were utilized under the commercial names “Aminoset”® and “Sacaka WS”®, respectively. “Aminoset”® consists of total organic matter 50%, organic carbon 20%, organic nitrogen 4%, and free amino acid 30%. On the other hand, “Sacaka WS”® contains organic matter 80% and fulvic acid 70%. The amino acid and fulvic acid doses in the root medium of lettuce were set at 100 mg/L and 40 mg/L, respectively. Additionally, “ERS” ® (Bioglobal Inc. Co.), a mycorrhizal mixture containing *Glomus intraradices, Glomus aggregatum, Glomus mosseae, Glomus clarum, Glomus monosporus, Glomus deserticola, Glomus brasilianum, Glomus etunicatum, and Gigaspora margarita* with a concentration of 1 × 10^4^ g^−1^, was applied to the seeds before sowing at a rate of 1000 spores seed^−1^ [45]. Furthermore, “Rhizofill” ® (NG-Biyoteknoloji Ltd. Co.), a mixture of Bacillus subtilis (1 × 109 ml-1), *Bacillus megaterium* (1 × 10^9^ ml^−1^), and *Pseudomonas fluorescens* (1 × 10^10^ ml^−1^), was used as a PGPR biostimulant at a dose of 1.0 ml L^−1^ [45]. “Adaga”® from Adaga company, containing 2.5% N-Acetyl-D-Glucosamine, was employed as chitosan at a dose of 300 µl/L in a hydroponic growing container. Lastly, “EkosolFarm”® (100% organic liquid vermicompost) from Ekosol Tarim company was used as vermicompost at a 2 ml/L dose. The treatments of the study were established as given in Table 1. In hydroponic lettuce cultivation, the nutrient solution was renewed every 10 days. Salt and biostimulant applications were also renewed.

**Table 1:** The study consisted of eight treatments, outlined as follows

Treatments	Explanation
Control (C)	The standard lettuce nutrient solution solely with mineral fertilizers, without any supplementation of biostimulants or salt addition
50 mM Salt (S)	The standard lettuce nutrient solution added 50 mM NaCl salt without supplementation of any biofertilizer
50 mM Salt + Amino Acid	The standard lettuce nutrient solution added 50 mM NaCl salt and supplemented aminoacid
50 mM Salt + PGPR	The standard lettuce nutrient solution added 50 mM NaCl salt and supplemented PGPR
50 mM Salt + Fulvic Acid	The standard lettuce nutrient solution added 50 mM NaCl salt and supplemented fulvic acid
50 mM Salt + Chitosan	The standard lettuce nutrient solution added 50 mM NaCl salt and supplemented chitosan
50 mM Salt + AMF	The standard lettuce nutrient solution added 50 mM NaCl salt and supplemented AMF.
50 mM Salt + Vermicompost	The standard lettuce nutrient solution added 50 mM NaCl salt and supplemented vermicompost.

*PGPR* Plant Growth Promoting Rhizobacteria, *AMF* Arbuscular mycorrhizal fungi

### Plant growth parameters

The harvested lettuce plants were individually weighed, and the total yield was expressed as kg m^‒2^ at the end of the 45 days of the growing period. The lettuce height*,* diameter, and circumferencewere measured using a ruler. The stem diameter was measured with a digital caliper as mm. The number of leaves per plant was recorded, and the leaf area was determined using a leaf area meter (Li-3100, LICOR, Lincoln, NE, USA), expressed as cm^2^ per plant. A digital penetrometer (Bareiss HPE-III-Fff, ABQ Industrial, USA) was utilized to quantify the firmness of lettuce, measured in kilograms. Chlorophyll content in the leaves was assessed using a leaf SPAD chlorophyll meter (SPAD-502, Minolta, Osaka, Japan). Leaf color values were digitally displayed on a portable digital handheld color spectrophotometer device (HunterLab, Virginia, USA) for the harvested lettuce leaves, and hue angle was calculated. Fresh weight (FW) of lettuce leaves was measured, followed by drying at 65 ºC for 24 h and reweighing (DW) to calculate the percentage of dry matter content (DW = 100 × DW/FW) [45].

### Lettuce antioxidant measurements

The methodology Spanos and Wrolstad [49] outlined determined total phenolic content with a modification. The quantification of total extracted phenolics was expressed in milligrams of Gallic acid (GA) equivalents, as determined by absorbance readings at 765 nm, utilizing a UV–visible spectrophotometer (UV-1700 Pharma Spec Shimadzu, Japan). The quantification of total flavonoid content in lettuce leaf samples using a UV–visible spectrophotometer (UV-1700 Pharma Spec Shimadzu, Japan) at 765 nm, as Quettier et al. [50] outlined. The total flavonoid substances were determined by a calibration prepared with standards. Vitamin C quantification was conducted employing the adapted procedure delineated by Elgailani et al. [51]. Basil leaves underwent homogenization using a high-speed blender, and a 5 mL basil extract was subsequently combined with 45 mL of 0.4% oxalic acid before filtration. The resulting filtrate, comprising 1 mL of extract and 9 mL of 2,6-dichlorophenolindophenol sodium salt, was subjected to transmittance measurement at 520 nm using a UV spectrophotometer.

### Mineral elements, sodium, and nitrate analysis

The concentrations of potassium (K), magnesium (Mg), calcium (Ca), iron (Fe), manganese (Mn), and zinc (Zn) in lettuce leaves were assessed using an atomic absorption spectrophotometer. Quarters of 3 individual plants from each replication were subjected to a drying process at 65 °C for 48 h, followed by grinding using a mill with a 20-mesh sieve. The resultant leaf powder underwent combustion in a furnace at 550 °C for 8 h, and the resulting ash was dissolved in 3.3% hydrochloric acid (HCl). Fe, Mn, Zn, and Cu Concentrations were determined through atomic absorption spectrometry in absorbance mode. In contrast, K, Ca, Mg, and Na concentrations were determined in emission mode [52]. The Kjeldahl and Barton methods determined leaf nitrogen and phosphorus levels [48]. The colorimetric determination of leaf nitrate accumulation in lettuce leaves was conducted through the transnitration of salicylic acid, as described by Cataldo et al. [53] and modified by Dasgan et al. [54].

### Antioxidative enzyme activities

The activity of antioxidant enzymes was assessed by extracting enzymes from 0.5 g of lettuce leaf tissue using a mortar and pestle, combined with 5 mL of extraction buffer containing 50 mM potassium-phosphate buffer at pH 7.6 and 0.1 mM disodium ethylenediaminetetraacetate. Following centrifugation of the homogenate for 15 min at 15.000 g, the supernatant fraction was utilized for enzyme assays. All enzyme extraction procedures were conducted at 4 °C, and activities were determined according to [55–57]. SOD activity was determined by monitoring the reduction of nitro blue tetrazolium (NBT) induced by superoxide radicals at a wavelength of 560 nm. A unit of SOD activity was defined as the enzyme amount required to inhibit 50% of NBT reduction by photochemical means. CAT activity was determined by monitoring the degradation rate of H_2_O_2_ at 240 nm. For this analysis, 50 mM phosphate buffer at pH 7.6 containing 0.1 mM EDTA, 0.1 ml of 100 mM H_2_O_2_, and enzyme extract were added to the reaction medium in a final volume of 1 ml. APX activity was determined by measuring ascorbate consumption at 290 nm. A unit of APX activity was defined as the enzyme amount required to metabolize one mole of ascorbate per minute. GR activity was determined by measuring the absorbance of nicotinamide adenine dinucleotide phosphate (NADPH) at 340 nm and its oxidation rate in the presence of the enzyme. The enzyme volume required to oxidize 1 mol of NADPH per minute was defined as 1 unit of GR activity.

### Determination of MDA (malondialdehyde) and relative water content (RWC)

Lipid peroxidation level was calculated based on the MDA (malondialdehyde) level determined using the thiobarbituric acid (TBA) reaction, following the method described by Heath and Packer [58] and modified according to Kusvuran and Yilmaz [59]. Absorbance was measured at 532 nm after centrifugation of the supernatant at 10.000 g for 10 min at 4 °C. Any non-specific absorption at 600 nm was subtracted from the values obtained. The proline content was quantified from aliquots of leaf crude extracts using the method outlined by Magne and Larher [60], improved by Dasgan et al. [61]. The collected leaf samples’ relative water content (RWC) was assessed on the day of harvest. Initially, the leaf samples were submerged in deionized water for 4 h. Subsequently, the turgor weights of the leaf samples were measured following this immersion period. Following these measurements, the leaf samples were dried in an oven at 65 °C for 48 h to determine their dry weight. The RWC of the leaves was calculated using the following equation [57]:

$$\text{RWC }({\%})\hspace{0.17em}=\hspace{0.17em}(\text{FW }-\text{ DW}) / (\text{TuW }-\text{ DW})\hspace{0.17em}\times \hspace{0.17em}100.$$

### Statistical analysis

The impacts of the treatments on morphological, physiological, and biochemical characteristics, as well as enzyme activities, were assessed utilizing the JMP statistical program (Version 7.0, Statistical Software, 2007). The means of the treatments were compared with the least significant difference (LSD) test at p ≤ 0.05 level.

## Results

### Lettuce yield and growth parameters

**Fig. 2:**
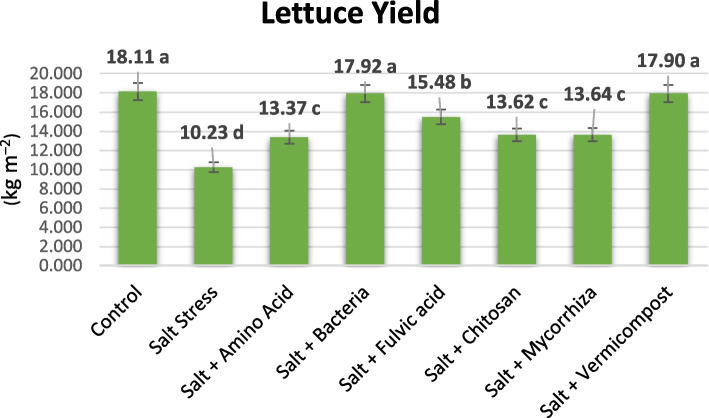
The yield of hydroponically grown lettuce under 50 mM saline water supplemented with biostimulants. There is no significant difference between means with the same letter in the same color histogram section

All plants treated with biostimulants exhibited higher yields than those subjected to salt stress. The control group yielded the highest leaf production at 18.11 kg m^‒2^. Under 50 mM salt stress conditions, bacterial and vermicompost biostimulants showed comparable results to the control plants within the same statistical group. The addition of vermicompost into the saline condition resulted in a total yield increase of 75% from 10.23 kg m^‒2^ to 17.92 kg m^‒2^, while PGPR supplementation yielded similarly 17.90 kg m^‒2^. Fulvic acid yielded a total of 15.48 kg m^‒2^, amino acid resulted in 13.37 kg m^‒2^, AMF addition resulted in 13.66 kg m^‒2^, and chitosan addition resulted in 13.62 kg m^‒2^ (Fig. 2). Yield increase rates according to salinity conditions were 51%, 31%, 34%, and 33%, respectively.

In terms of the lettuce weight, all biostimulant supplements significantly enhanced lettuce weight compared to that under saline conditions (230 g). However, control (407.7 g), PGPR (403.5 g), and vermicompost (403.6 g) treatments were found to belong to the same statistical group regarding lettuce weight (Table 2). According to the plant growth data, biostimulants applied to lettuce plants induced significant changes in plant height under salt stress. Measurements indicate that all applied biostimulants statistically resulted in taller plant height than salt application alone. All biostimulant applications and controls are grouped in the plant height statistical analysis. Salt + PGPR (45.50 cm), salt + vermicompost (44.65 cm), salt + chitosan (43.30 cm), and salt + fulvic acid (41.40 cm) treatments resulted in better plant width compared to control (40.80 cm) plants. The salt treatment produced the most petite plant circumference, with a value of 65.20 cm, and statistically, the lowest value was observed in the salt treatment alone. Salt + vermicompost was the most effective treatment regarding plant circumference, with a value of 76.55 cm. All biostimulant treatments resulted in more leaves than the control (33.75 leaf) and salt treatment (23.12 leaf) groups. The highest number of leaves was observed in the salt + vermicompost treatment (41.50 leaves), which produced more leaves than the control group. Control plants exhibited the largest leaf area (5340 cm^2^ per plant), while the lowest leaf area was observed in the salt treatment group (2946 cm^2^ per plant). Among the biostimulants, the salt + vermicompost treatment had a leaf area of 4748 cm^2^ per plant, and bacterial treatments had a leaf area of 4703 cm^2^ per plant, making them the most successful treatments with leaf areas closest to the control plants. As anticipated, control plants exhibited the most prominent stem diameter (17.41 mm). Among the biostimulant treatments, only the salt + vermicompost application (16.93 mm) fell within the same group as the control plants. All other biostimulant treatments yielded higher results than the salt stress condition (11.43 mm).

**Table 2:** Plant growth parameters of hydroponically grown lettuce under 50 mM saline water supplemented with biostimulants

Treatments	Plant weight (g)	Plant Height (cm)	Plant Diameter (cm)	Plant Circumference (cm)	No of Leaf per Plant	Leaf Area (cm^2^ plant^‒1^)	Stem Diameter (mm)
Control	407.6 a	22.15 a	40.40 cd	68.80 ac	33.75 b	5340 a	17.41 a
Salt	230.5 d	20.05 b	29.80 e	65.20 c	23.12 c	2946 f	11.43 d
Salt + Aminoacid	307.1 c	21.55 ab	32.90 e	73.55 ab	36.62 ab	4437 c	15.00 c
Salt + PGPR	403.5 a	22.35 a	45.50 a	67.70 bc	39.12 ab	4703 b	14.66 c
Salt + Fulvic acid	348.6 b	20.95 ab	41.80 bd	67.05 bc	36.50 ab	3760 d	16.04 b
Salt + Chitosan	306.7 b	21.35 ab	43.30 ac	68.10 bc	38.12 ab	3560. e	16.22 b
Salt + AMF	307.3 c	22.25 a	39.05 d	68.85 ac	40.00 ab	3891 d	16.19 b
Salt + Vermicompost	403.6 a	22.05 a	44.65 ab	76.55 a	41.50 a	4748 b	16.93 a
*P*	0.001	0.01	0.0001	0.013	0.0002	0.0001	0.0001
LSD_0.05_	90.25	1.60	3.62	8.15	7.19	155.0	0.66

There is no significant difference between means with the same letter in the column

*PGPR* Plant Growth Promoting Rhizobacteria, *AMF* Arbuscular mycorrhizal fungi, *LSD* The least significant difference between the means (*p* ≤ 0.05)

### Lettuce quality properties

Lettuce head firmness was analyzed, revealing an increase in firmness due to salt application. Salt + biostimulants generally increased firmness, except for chitosan application (Table 3). Firmness decreased in the control group (2.38 kg cm^2^). The highest firmness was observed in the salt + AMF treatment (2.73 kg cm^2^), and all biostimulants were within the same statistical group as the salt treatment (2.51 kg cm^2^). The highest percentage of dry matter was calculated in the salt treatment (5.11%), followed by salt + fulvic acid (4.96%) and salt + PGPR (4.77%), which were in the same group as the salt treatment and higher than the control. Dry matter in the remaining treatments was in the same statistical group as the control (4.18%). Salt application has been shown to increase nitrate content in lettuce leaves, while Fulvic acid, PGPR, chitosan, and vermicompost applications have significantly decreased nitrate by 68%, 11%, 9% and 12%, respectively. The highest chlorophyll content was observed in the salt + PGPR application (34.97). All biostimulant treatments produced higher chlorophyll than both the control and salt treatments. The chlorophyll content of salt-treated plants (31.66) and control plants (30.51) fell within the same statistical group. The lowest Hue value recorded was 106.36 in the salt treatment, indicating a color closest to yellow on the scale. Treatments supplemented with biostimulants exhibited higher Hue values compared to the salt treatment, and these biostimulant-treated samples fell within the same statistical group as the control. Consequently, it was observed that treatments other than salt exhibited hues closer to green than those of the control group.

**Table 3:** Firmness, dry matter, chlorophyll, leaf color (Hunter hue angle) of hydroponically grown lettuce under 50 mM salt and biostimulants

Treatments	Firmness (kg cm^‒2^)	Dry Matter (%)	Nitrate (mg kg FW^‒1^)	Chlorophyll-SPAD	Hue Angle (°)
Control	2.38 b	4.18 c	534 b	30.51b	108.27 a
Salt	2.51 a	5.11 a	570 a	31.66 b	106.36 b
Salt + Aminoacid	2.57 a	4.33 c	588 a	32.97 ab	107.69 ab
Salt + PGPR	2.47 a	4.77 ab	511 cd	34.97 a	107.68 ab
Salt + Fulvic acid	2.51 a	4.96 a	462 e	33.00 ab	108.32a
Salt + Chitosan	2.04 b	4.31 c	519 bc	33.19 ab	107.37 ab
Salt + AMF	2.73 a	4.34 bc	591 a	32.99 ab	107.87 a
Salt + Vermicompost	2.51 a	4.05 c	501 d	33.04 ab	108.82 a
*P*	0.0305	0.0001	0.0001	0.0410	0.0436
LSD_0.05_	0.3578	0.43	19,02	2.69	1.494

There is no significant difference between means with the same letter in the column

*PGPR* Plant Growth Promoting Rhizobacteria, *AMF* Arbuscular mycorrhizal fungi, *LSD* The least significant difference between the means (*p* ≤ 0.05), *FW* Fresh weight

### Lettuce antioxidant properties

Plants subjected to salt stress produced higher levels of total phenolic compounds than the control group. However, plants treated with biostimulants exhibited an even higher accumulation of phenolic substances than the control group and those treated solely with salt. Specifically, treatments containing salt + vermicompost, salt + PGPR, and salt + fulvic acid were characterized by the highest total phenolic content (Table 4). Total flavonoids were not higher than salt. Salt, salt + amino acid, and salt + PGPR treatments exhibited higher total flavonoid levels. Salt + vermicompost followed them in fourth place. On the other hand, the flavonoid level of salt + fulvic acid was determined to be the lowest. Regarding vitamin C content, the highest level was recorded in the salt treatment (19.34 mg100g FW^‒1^), whereas the lowest vitamin C content was determined in the control group (15.21 mg100g FW^‒1^). According to the statistical analysis, the closest result to the salt treatment was obtained from the salt stress + vermicompost treatment (18.96 mg100g FW^‒1^). It was indicated that the plants treated with biostimulants formed an intermediate value between the control group and the salt treatment.

**Table 4:** Antioxidant contents of hydroponically grown lettuce under 50 mM saline water by the biostimulant supplements

Treatments	Total Phenol (mg GA 100 g FW^‒1^)	Total Flavonoid (mg RU 100 g FW^‒1^)	Vitamin C (mg 100 g FW^‒1^)
Control	19.24 e	24.36 d	15.21 f
Salt	20.82 d	26.53 a	19.34 a
Salt + Aminoacid	22.34 b	26.50 a	16.70 d
Salt + PGPR	23.85 a	26.13 ab	17.19 c
Salt + Fulvic acid	24.51 a	22.26 e	16.19 e
Salt + Chitosan	21.27 cd	24.36 d	16.57 d
Salt + AMF	21.63 bc	25.20 c	17.47 c
Salt + Vermicompost	24.61 a	26.03 b	18.96 b
*P*	0.0001	0.0001	0.0001
LSD_0.05_	0.8042	0.4270	0.3345

There is no significant difference between means with the same letter in the column

*PGPR* Plant Growth Promoting Rhizobacteria, *AMF* Arbuscular mycorrhizal fungi, *LSD* The least significant difference between the means (*p* ≤ 0.05), *FW* Fresh weight, *GA* Gallic acid, *RU* Rutin

### Macro and micro mineral element concentrations of lettuce

Macro mineral element concentrations were consistently maintained within the nutrient reference ranges established for lettuce plants outlined [48] (Table 5). However, amino acids and PGPR applications contained higher nitrogen levels than salt and control treatments. On the other hand, the nitrogen concentrations of fulvic acid, chitosan, and vermicompost were found to be lower than those of the salt application. However, all nitrogen concentrations in the trial remained within the reference range of 2–4% for lettuce plants. The plants did not suffer from nitrogen deficiency. The phosphorus concentration was highest in the control and lowest in the salt application. Biostimulant applications contained higher phosphorus levels compared to the salt application. The potassium concentration was highest in the control and lowest in the salt application. The biostimulant applications significantly increased the potassium content in lettuce leaves compared to salt treatment. The K content was highest in the salt + vermicompost treatment. A similar situation was observed for Ca and Mg, where biostimulant applications were found to increase the Ca and Mg concentration under salinity conditions.

**Table 5:** Macronutrient concentrations of hydroponically grown lettuce under 50 mM saline by the biostimulant supplements (%)

Treatments	N	*P*	K	Ca	Mg
Control	4.73 b	0.56 a	7.16 ab	1.07 a	0.63 a
Salt	4.29 cd	0.27 d	3.97 d	0.52 d	0.35 d
Salt + Aminoacid	5.51 a	0.36 c	7.01 bc	0.69 bc	0.45 c
Salt + PGPR	5.25 a	0.53 a	7.23 bc	0.81 b	0.43 cd
Salt + Fulvic acid	4.22 d	0.28 d	6.48 c	0.60 cd	0.36 d
Salt + Chitosan	4.21 d	0.37 c	7.70 ab	0.60 cd	0.54 b
Salt + AMF	4.63 bc	0.43 b	7.40 ac	0.61 cd	0.43 cd
Salt + Vermicompost	3.32 e	0.54 a	8.31 a	0.79 b	0.53 b
*P*	0.001	0.001	0.001	0.001	0.001
LSD_0.05_	0.34	0.05	0.94	0.14	0.08

There is no significant difference between means with the same letter in the column

*PGPR* Plant Growth Promoting Rhizobacteria, *AMF* Arbuscular mycorrhizal fungi, *LSD* The least significant difference between the means (*p* ≤ 0.05)

**Fig. 3:**
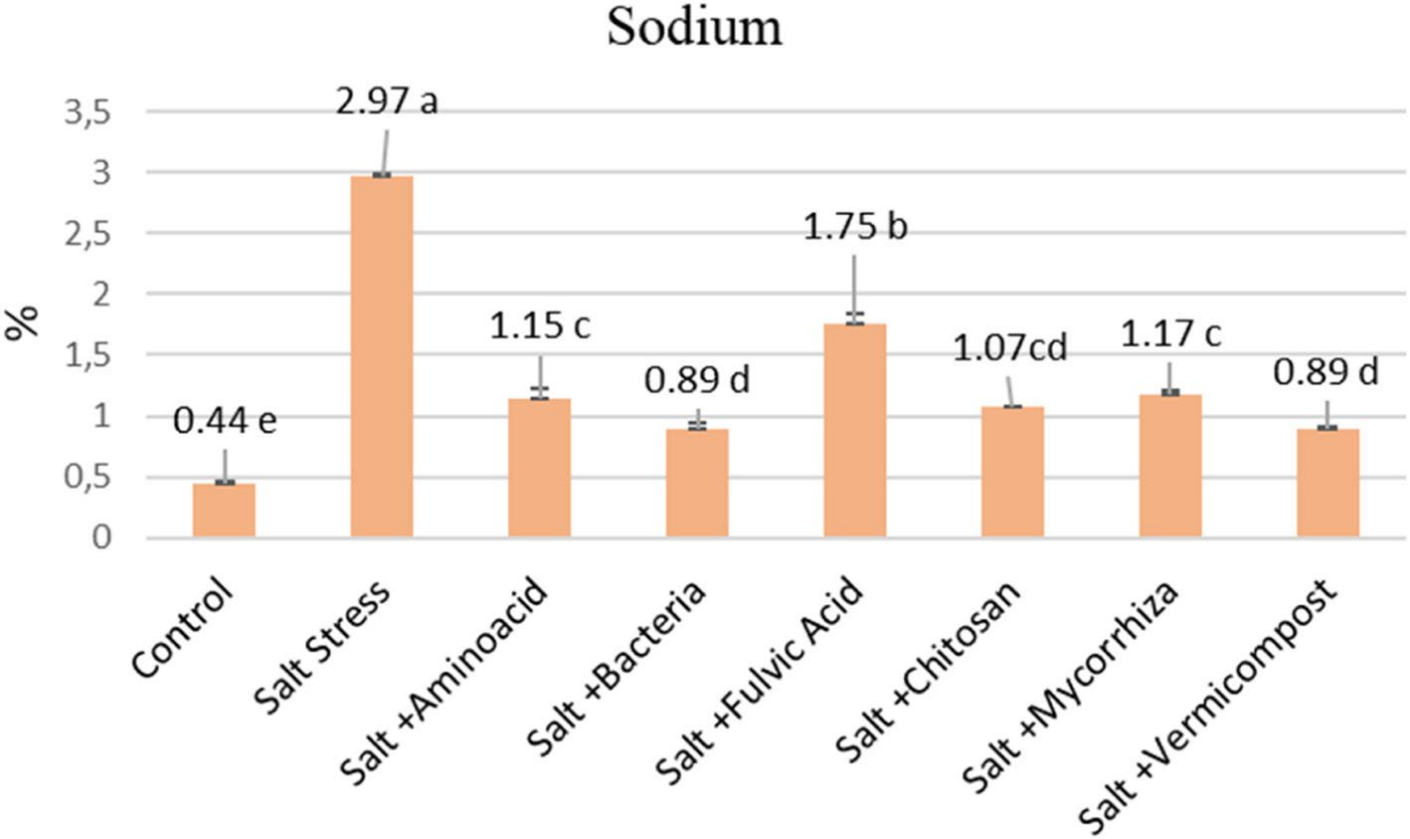
Effects of biostimulants on leaf Na concentration of hydroponically grown lettuce under 50 mM salinity water by the biostimulant supplements. There is no significant difference between means with the same letter in the same color histogram section

In terms of micronutrient content (Fe, Mn, Zn and Cu) in lettuce leaves, it was observed that biostimulants increased the concentrations of micronutrients compared to salt stress. However, the PGPR application notably surpassed even the control in all micronutrients, exhibiting the highest concentrations (Table 6). All the biostimulant applications have significantly reduced Na content by between 75 and 233%. A significant reduction in Na was observed with PGPR and vermicompost applications, while the fulvic acid treatment noted the most minor decrease (Fig. 3).

**Table 6:** Micronutrient concentrations of hydroponically grown lettuce under 50 mM salt by the biostimulant supplements (mg kg^‒1^)

Treatments	Fe	Mn	Zn	Cu
Control	93.54 b	32.78 b	52.81 b	13.80 a
Salt	43.85 f	15.14 d	23.24 e	2.15 d
Salt + Aminoacid	64.41 d	26.28 c	46.18 c	3.29 d
Salt + PGPR	110.09 a	43.15 a	74.54 a	15.5 a
Salt + Fulvic acid	51.76 e	19.82 d	38.66 d	6.44 c
Salt + Chitosan	62.65 d	25.98 c	42.37 cd	8.56 b
Salt + AMF	42.29 f	15.96 d	22.56 e	7.66 bc
Salt + Vermicompost	73.20 c	31.52 b	22.22 e	6.10 c
*P*	0.001	0.001	0.001	0.001
LSD_0.05_	5.78	4.85	6.29	1.92

There is no significant difference between means with the same letter in the column

*PGPR* Plant Growth Promoting Rhizobacteria, *AMF* Arbuscular mycorrhizal fungi, *LSD* The least significant difference between the means (*p* ≤ 0.05), *FW* Fresh weight

### Stomatal conductance, RWC and membrane injury

In lettuce leaves grown hydroponically, 50 mM NaCl salinity caused a decrease in stomatal conductance by 205% compared to the control. However, biostimulant applications significantly ameliorated this adverse effect of salt stress. Accordingly, biostimulants increased stomatal conductance by 58% to 189%. Among the biostimulants the highest increase rate was recorded in the chitosan application (Table 7). RWC used as an indicator of leaf water status, decreased by 22% in plants under salt stress. RWC, which decreased under saline conditions, increased by 9% to 108% with biostimulant applications. The highest increase rate was recorded in the amino acid biostimulant application. The injury rate of biomembranes due to salt stress was 39% compared to control. Biostimulant applications played a positive role in reducing this damage by 14% to 58%. The most effective treatments were PGPR and vermicompost, with 58%, while fulvic acid was the least effective.

**Table 7:** Effects of the biostimulants on stomatal conductance, RWC, and membrane injury

Treatments	Stomatal Conductance (mmolm^‒2^s^‒1^)	RWC (%)	Membran injury (%)
Control	145.69 a	15.45 b	2.91e
Salt	47.80 g	12.65 b	7.54 a
Salt + Aminoacid	90.63d	26.30 a	4.62 d
Salt + PGPR	80.56 ef	19.76 ab	3.19 e
Salt + Fulvic acid	86.52 de	16.96 b	6.46 b
Salt + Chitosan	138.18 b	16.49 b	5.96 c
Salt + AMF	111.12 c	14.15 b	6.10 bc
Salt + Vermicompost	75.39 f	13.78 b	3.19 e
*P*	6.15	11.05	
LSD_0.05_	0.001	0.05	

There is no significant difference between means with the same letter in the column

*PGPR* Plant Growth Promoting Rhizobacteria, *AMF* Arbuscular mycorrhizal fungi, *RWC* Relative water content, *LSD* The least significant difference between the means (*p* ≤ 0.05)

### Lipid peroxidation, antioxidant enzyme activities, and proline content

**Fig. 4:**
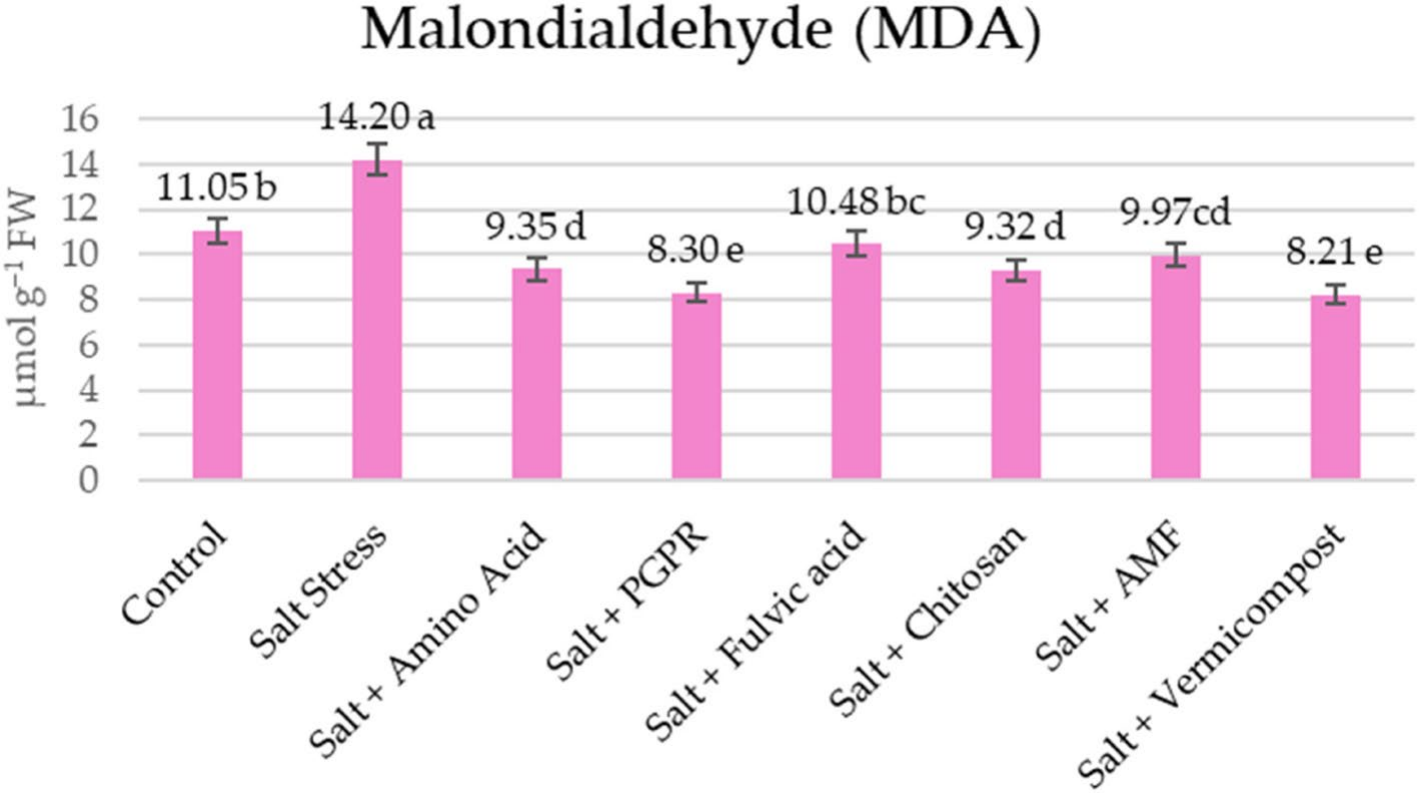
Effects of biostimulants on lipid peroxidation of lettuce grown hydroponically under 50 mM NaCl saline water

**Fig. 5:**
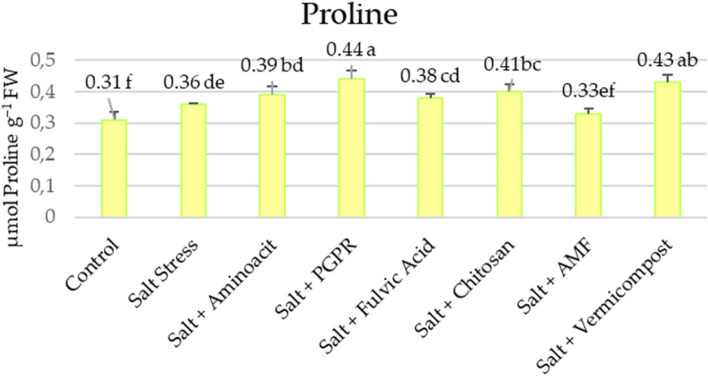
Effects of biostimulants on proline content of lettuce grown hydroponically under 50 mM NaCl saline water

Cellular damage was estimated by measuring lipid peroxidation in terms of MDA content. Significant membrane damage was observed under salt conditions (with a 29% increase) compared to control. However, the treatment with biostimulants showed lower MDA values, resulting in statistically significant cellular protection compared to salt-stressed plants. Remarkably, plants treated with PGPR and vermicompost demonstrated the lowest MDA content in the presence of salt stress, with decreases of 41.5% and 42.2%, respectively (Fig. 4). Figure 5 shows that salt stress also increased the contents of osmoprotectants such as proline by 16.13% compared to control. Additionally, biostimulants maximizedthe proline contents in the lettuce leaves under salt stress. The highest increase in proline content (0.44) was observed in plants treated with PGPR under salinity imposition. The most effective treatments were PGPR and vermicompost, which increased proline contents by 22.2% and 19.4%, respectively, compared to the salt treatment.

**Fig. 6:**
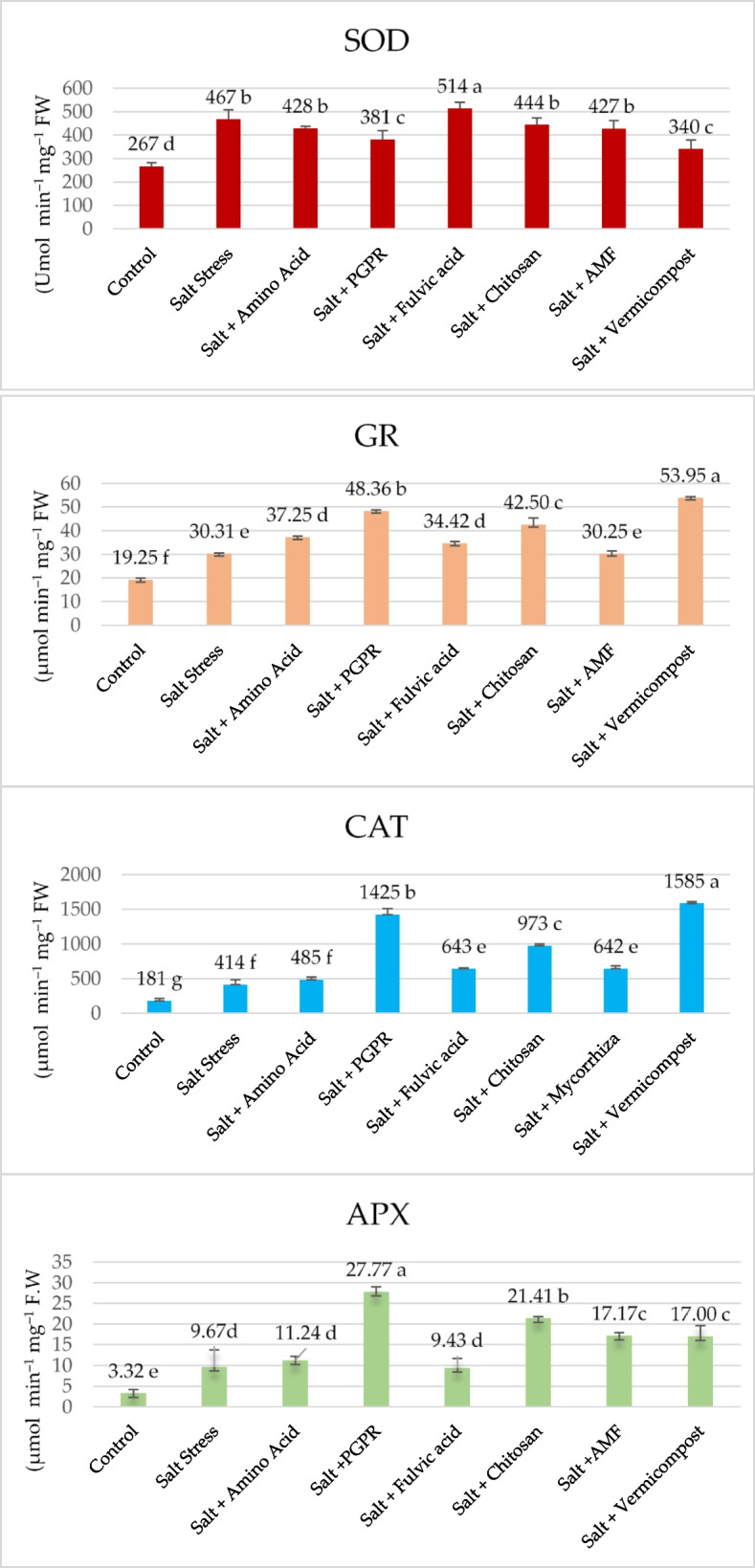
Effects of biostimulants on antioxidant enzyme activities of hydroponically grown lettuce under 50 mM NaCl saline water

In lettuce, under 50 mM salt conditions, SOD (157.37 U min^‒1^ mg ^‒1^FW), CAT (414.12 μmol min^‒1^ mg^‒1^ FW), GR (30.31 μmol min^‒1^ mg^‒1^FW), and APX (9.67 μmol min^‒1^ mg^‒1^FW) enzyme activities increased by 98%, 181%, 19%, and 3%, respectively, compared to control plants (Fig. 6). Under salt stress, the activity of the SOD enzyme in plants treated with biostimulants was lower than in plants that were not stressed, but it increased by 43% when plants were exposed to PGPR. In this study, they were using biostimulants allowed for an increase of 233%, 98%, and 120% in CAT, GR, and APX enzyme activities compared to salt-treated plants. When biostimulants were evaluated among themselves, the highest increase in these enzyme activities was determined in PGPR and vermicompost applications (433% and 502%, 151% and 180%, and 736% and 412% increase).

## Discussion

In response to salt stress, plants develop defense mechanisms such as ion homeostasis, osmotic adjustment, and enhancement of antioxidant defense systems. However, prolonged stress may overwhelm these mechanisms [23]. As a novel strategy,exogenously applied biostimulants protect plants from adverse conditions, promoting sustainable agricultural production. Scientists, growers, and the fertilizer industry have already adopted this strategy. Numerous fertilizer companies and start-ups are striving to develop biostimulant products to alleviate the adverse impacts of abiotic stresses, such as drought and salinity. This study assessed the effects of various biostimulants on hydroponically grown lettuce plants under 50 mM salt stress. Consistent with our initial hypotheses, the agronomic and physiological results demonstrated that biostimulants can effectively alleviate the adverse impacts of saline water on hydroponic culture. Below, we discuss plants’ physiological mechanisms to tolerate salt stress conditions and how biostimulants enhance this tolerance.

The elevated levels of NaCl in irrigation water can lead to several detrimental effects, including [11, 62] 1) Reduced water uptake and leaf turgor due to osmotic stress, 2) Toxicity of Na and Cl ions within various plant tissues, 3) Potential nutritional imbalances. It is well-documented in the literature that lettuce growth and yield are hampered by salt stress [32, 63]. As observed in our study, salt stress induces various morphological and physiological changes, thereby restricting lettuce growth, yield, and quality. The biostimulant applications alleviated the detrimental effects of salt on plant weight, height, diameter, circumference, leaf number, firmness, leaf area, and yield. Plants treated with biostimulants exhibited enhanced growth, stomatal conductance, relative water content, antioxidants, proline as osmoprotectant, and photosynthetic pigments compared to plants grown under saline conditions. The biostimulants used here mitigated the salt-induced damage by reducing the accumulation of Na and maintaining ions.Using biostimulants is an effective strategy for mitigating the adverse effects of salinity stress on plant health. Biostimulants enhance plant vitality through various mechanisms, including hormonal stimulation, siderophore synthesis, exopolysaccharide secretion, osmoprotectant accumulation, ion exchange, and activating antioxidant enzymes—homeostasis, and increasing the activities of antioxidant enzymes (SOD, APX, CAT, GR).

Using biostimulants is an effective strategy for mitigating the adverse effects of salinity stress on plant health. Biostimulants enhance plant vitality through various mechanisms, including hormonal stimulation, siderophore synthesis, exopolysaccharide secretion, osmoprotectant accumulation, ion exchange, and activating antioxidant enzymes [25, 27].

### Regulatory effects of biostimulants on stomatal conductance, chlorophyll content, and water status about plant growth and yield of lettuce under salt stress

In response to salinity-induced water stress (osmotic stress), plants generate ABA, which primarily regulates plant water balance by initiating stomatal closure and decreasing transpiration and water uptake [64]. It has been reported that plants close their stomata under salinity stress to minimize water loss through transpiration and limit the uptake of Na and Cl ions through the roots by xylem flux [14]. However, this adjustment leads to a decline in intercellular CO_2_ concentration and carbon assimilation, resulting in diminished photosynthetic rates and restricted growth [27, 65]. In our study, plants supplemented with biostimulants exhibited higher stomatal conductance (58–189%) than salt-stressed plants (Table 6). Under salt stress, a reduction in chlorophyll content has been observed, attributed to the increased activity of the enzyme chlorophyllase [66]. Therefore, our results showed that lettuce plants treated with biostimulants exhibited increased photosynthetic pigment-chlorophyll (4–10%), mineral nutrient uptake (15–109%), and better water status (RWC) (9–107%) to maintain photosynthesis, thereby preventing restriction on plant growth and yield. According to the study, lettuce yield decreased by 44% under 50 mM salinity. When biostimulants were added under saline conditions, lettuce yield increased by 30% to 75% (Fig. 1). The highest increase in lettuce yield under saline conditions was observed with PGPR and vermicompost applications. The presence of biostimulants significantly mitigated the inhibition of lettuce plant growth caused by salt stress. The biostimulants have increased significantly plant growth parameters such as weight (%33–75), height (4.5–11%), diameter (6–11%), circumference (3–17%), number of leaves (55–75), stem diameter (31–48%, (leaf area (61–80%) (Table 2) [10]. Moncada et al. [27] reported that incorporating a bacterial biostimulant into the nutrient solution of floating-grown lettuce effectively alleviated the impact of 20 mM salt stress. Remarkably, nearly all morphological, physiological, and yield parameters assessed in the plants subjected to salt stress and treated with the bacterial biostimulant were similar to or even surpassed those of control plants. Consequently, the detrimental effects of salinity were wholly overcome. Parihar et al. [67] demonstrated that the inoculation of arbuscular AMF alleviated the adverse impacts of salinity on lettuce plants. This was attributed to increased nutrient uptake, antioxidant and enzyme activities, stomatal conductance, RWC, proline accumulation, and reduced cellular electrolyte leakage. MDA ultimately enhanced biomass production, chlorophyll synthesis, yield, and growth characteristics.

The observed % decrease in relative water content (RWC) by 22% under salt stress compared to the control in this study suggests that the plants were experiencing osmotic stress [10]. The biostimulants positively influenced plant-water relations. The biostimulants increased RCW by 9–107% (Table 7) [68].

### Enhancing effects of biostimulants on minerals nutrients under salt stress

Salt-affected plants accumulate excessive Na+ and Cl− ions, which are absorbed more quickly than essential ions. This leads to ionic toxicity andincreases the disruption of nutrient balance by interaction with Na and Cl ions, disrupting the uptake and transportation of importantions such as N, P, K, Ca, Mg, Fe, Mn, Zn, B, and Mo [14]. The ability of plants to tolerate salt largely depends on managing toxic Na accumulation and its distribution within plant parts [69]. The higher Na causes Ca and K uptake restriction, distorting cell functioning, such as photosynthetic capacity, antioxidant enzyme activities, protein biosynthesis, and hormone metabolism, thereby reducing plant growth and yield [70]. Sustaining nutrient equilibrium and regulating the Na/K and Na/Ca ratios are paramount for plant growth and survival in saline environments [71]. In our study, salt-stressed lettuce plants exhibited elevated Na levels. They diminishedK, Ca, and Mg levels, indicating a disturbance in ion homeostasis and subsequent reduction in plant growth and yield (Table 4). However, supplementation with biostimulants led to increased concentrations of macro nutrients P (4–97%), K (36–109%), Ca (15–56%) and Mg (23–54%) and micro nutrients Fe (18–150%), Mn (5.5.-185%), Zn (66–222%), Cu (53–620%) while decreasing Na (41–70%) levels (Table 5 and 6 and Fig. 3).

For hydroponically grown lettuce plants, applying biostimulants enhances the availability and uptake of essential nutrients, facilitating their incorporation into the chlorophyll biosynthetic pathway. The high levels of chlorophyll and mineral nutrients promote increased rates of photosynthesis, evaporative transpiration, intercellular CO_2_ concentration, and chlorophyll content, ultimately resulting in an elevated net assimilation rate [72].

The application of biostimulants mitigated the Lettuce plant water status, which increased osmotic adjustment due to elevated levels of mineral osmolytes such as K, Ca, Mg, P, and organic-osmolyte-proline. Generally, synthesis and accumulation of cellular compatible solutes, commonly known as osmolytes or osmoprotectants, help plants overcome osmotic stress, termed osmotic adjustment [73].Osmoprotectants encompass a variety of inorganic ions such as K, Ca, and Mg and organic solutes such as aminoacids, sugars, and carbonhydrates that reduce the osmotic potential by increasing their concentrations, thereby enhancing cellular water retention during water stress [74]. In this study, biostimulants regulated osmotic adjustment by enhancing the uptake and accumulation of mineral elements under salt stress. Similarly, the production of organic osmolytes, such as proline, has also been promoted by biostimulants. Minerals and proline acted as osmotic protectants. Cell turgor maintenance is crucial as it directly influences stomatal arrangement, affecting photosynthetic capacity [73]. The following mechanisms can explain the maintenance of cell turgor pressure under biostimulant application: (i) enhanced water uptake, (ii) increased accumulation of osmolytes, (iii) improved nutrient uptake, (iv) strengthened cell walls, and (v) activation of stress-responsive pathways involving water and ion transport, osmolyte synthesis, and antioxidative defense. These combined effects help plants sustain turgor pressure under salt stress, preventing cell water loss [72, 73].

Benazzouk et al. [75] demonstrated that applying vermicompost to salt-treated tomato plants helps maintain their net photosynthesis, limits Na translocation from roots to shoots, and enhances osmotic adjustment primarily through proline synthesis, accumulation of various nutrients N, P, K, Ca, Mg, which may contribute to salt resistance. Beykkhormizi et al. [68] cultivated bean plants under 20–80 mM NaCl stress and applied vermicompost to alleviate salt-induced damage. Applying vermicompost significantly increased the K and Ca concentrations in leaf and root tissues while reducing Na uptake under saline conditions.

### Biofertilizers enhance osmoprotectant and oxidative defense system in lettuce under salinity

Numerous plant species synthesize organic compatible solutes in salt stress, including amino acids such as proline and glycine betaine [73]. The buildup of these substances creates the osmotic potential required for water uptake while maintaining cellular metabolism [14]. Proline, a low molecular weight water-soluble amino acid, is recognized as one of the primary osmoregulators/osmoprotectants responsible for regulating plant salinity toleranceto maintain cellular–water relations through its accumulation. Proline plays a crucial role in osmotic adjustment and offers protective functions in salt-treated plants, including scavenging free radicals and safeguarding intracellular structures against NaCl-induced oxidative stress [14]. Our results indicate that biostimulants enhanced the proline content in lettuce plants by up to 22% under salt stress conditions (Fig. 5), facilitating better cellular osmotic adjustment. Biostimulants induce proline accumulation in tissues subjected to osmotic stress such as salinity, promoting osmotic homeostasis and combating oxidative stress [76]. Al Huqail1 et al. [72] reported that in response to salt stress, proline, and soluble sugars act as compatible solutes that reduce the water potential of the plant, thereby establishing a gradient favorable for water uptake and restoring cellular turgor. Additionally, proline functions as an antioxidant, signaling molecule, and protective agent, safeguarding biomolecules from the damaging effects of salt-induced dehydration.

Under salt stress, plants initiate a response by generating reactive oxygen species (ROS), which serve as signaling molecules while also causing damage to root and shoot tissues by disrupting enzyme function and cell wall integrity [12]. The ROS can also cause damage to DNA, lipids, and proteins. Simultaneously, ROS induces chlorophyll breakdown and membrane lipid peroxidation, decreasing membrane fluidity and selectivity [14, 16]. To defend against oxidative stress, plants require an effective antioxidant system that includes non-enzymatic and enzymatic antioxidants. When applied exogenously through priming, irrigation, soil addition, or foliar spraying, biostimulants can reduce ROS-induced oxidative damage under salt stress. They enhance salt tolerance by strengthening the antioxidant defense mechanism and minimizing oxidative damage at the cellular level [77].

Biostimulants are thought to influence ROS homeostasis by preventing metals from auto-oxidizing, reducing available electrons for ROS production, and enhancing antioxidant activity to scavenge ROS. Their exogenous use is being explored to develop plant salt tolerance [78]. Results of this study showed that biostimulants efficiently activate enzymatic antioxidant systems and reduce the harmful effects of salinity; nevertheless, PGPR and vermicompost treatment were more effective than the other treatments. In this study, the supplementation with biostimulants resulted in increased activities of APX (16–187%), CAT (17–283%), and GR (16–78%) enzymes in salt-stressed lettuce plants (Fig. 6). SOD activity increased by only 10% with fulvic acid. The findings of this investigation were consistent with the results reported by [78–80]. Enhanced enzyme activity has been documented to enhance the growth of stressed plants by protecting chloroplasts and other organelle structures where vital biological processes occur [64]. Rakkammal et al. [81] reported that biostimulated lettuce plants under salt stress exhibited significantly higher activity of guaiacol peroxidase (GPX) and catalase (CAT) compared to non-biostimulated plants, highlighting the importance of these enzymes in the removal of hydrogen peroxide. These studies similarly observed notable variations in antioxidant enzyme activity after administering biostimulant therapy to plants experiencing salt stress. Biostimulant treatments enhanced the concentrations of antioxidant metabolites and enzyme activity in chloroplasts under salt stress conditions, which corresponded to the biostimulant’s ability to decrease the levels of MDA in these cellular structures [82].

Lipid peroxidation destroys the integrity of cell membranes, resulting in cell death over time. MDA, a biomarker of cellular toxicity, results from lipid peroxidation under oxidative stress conditions [83]. Our study observed an elevation (29%) in MDA content under salt stress conditions (Fig. 4). As indicated in our research, the application of biostimulants considerably lowered MDA content by 26–42%. The most effective reduction was with vermicompost, which might affect the metabolism and be responsible for the increase in photosynthesis [84]. The administration of the biostimulants significantly reduced salt-induced oxidative stress, as evidenced by decreased MDA levels. This finding indicates that biostimulants contain antioxidant compounds that act as ROS scavengers against salt-induced H2O2, thereby protecting and stabilizing the cellular membranes of lettuce leaves, maintaining their fluidity, and reducing MDA levels [72]. It is believed that the positive effects of biostimulants are attributable to their antioxidant properties, which could prevent lipid peroxidation in cell membranes during environmental stress [10, 85, 86]. Zuzunaga-Rosas et al. [87] reported that applying a complex mixture of amino acids and oligopeptides significantly reduced MDA levels in lettuce plants subjected to salt treatments ranging from 50 to 150 mM NaCl. This finding confirms the role of biostimulants as protective agents against oxidative damage. Adequate stress tolerance can be achieved by detoxifying reactive ROS, primarily facilitated by enzymatic and non-enzymatic antioxidants [88–90]. These antioxidants enhance plant survival under stressful conditions by protecting them from oxidative damage [14]. In our study, total phenols, one of the antioxidants, has been increased by 4–18% by the biostimulants.

In this study, fulvic acid, PGPR, chitosan, and vermicompost applications significantly decreased the nitrate of lettuce under saline conditions, which was 462–519 mg kg^−1^ FW (Table 3) [22, 48, 54]. Nitrate accumulation in lettuce can exhibit significant variability depending on the variety and the conditions under which it is cultivated. Research indicates that nitrate levels in curly lettuce have been recorded within a broad range, from 16 to 3400 mg kg^−1^ FW, with an average of 1601 mg kg^−1^ FW [91]. In our investigation, nitrate concentrations remained well below the thresholds deemed harmful to human health. Notably, the European Commission (EC Reg. No. 1258/2011) set the commercialization threshold for protected-grown lettuce cultivated under cover from October to March, is established at 5000 mg kg − 1 FW, a limit that our study did not exceed.

The efficacy of PGPR in improving crop resilience against salinity may stem from diverse mechanisms. These mechanisms include alterations in phytohormone levels, reinforcement of antioxidant defenses, an increase of osmolyte synthesis, and activation of ACC (1-aminocyclopropane-1-carboxylate) deaminase activity [27, 92]. Ethylene levels elevate in plants experiencing salt stress, adversely impacting photosynthesis and stomatal conductance [93]. Certain *Bacillus* spp. possess the capacity to regulate ethylene production in roots via the enzyme ACC-deaminase [94], offering a potential avenue for mitigating the adverse effects of salt stress on plant physiology. Benazzouk et al. [75], reported that vermicompost ameliorated the adverse effects of salinity due to its abundant nutrients, plant hormones such as cytokinin and gibberellic acid, auxin for better plant growh under salt stress. Gibberellic acid reduced electrolyte leakage and significantly improved cell membrane stability [68].

## Conclusion

In agricultural crop production, the use of high-quality water is increasingly restricted. In vegetable cultivation, particularly in greenhouse and soilless farming, the use of good-quality clean water is of paramount importance. However, the scarcity of water resources often requires the use of saline groundwater for agricultural irrigation. This study demonstrates that the hydroponic cultivation of lettuce, a salt-sensitive vegetable, in the case of poor-quality saline water necessitates significant reliance on biostimulants as a crucial solution. It has been shown that plants can effectively cope with saline conditions through biostimulants without adversely affecting yield and crop quality. Specifically, PGPR (1.0 ml L^−1^), vermicompost (2 ml L^‒1^) and fulvic acid (40 mg L^‒1^) biostimulants have emerged as prominent solutions in saline water conditions, making them recommendable for hydroponic lettuce producers. This study demonstrates that biostimulants can substantially enhance the sustainable production of hydroponic lettuce by increasing plant tolerance to salt stress, improving nutrient uptake, and boosting crop yield and quality. The biostimulants are affordable, environmentally friendly, and green treatment for mitigating the detrimental consequences of salt stress. It is anticipated that hydroponic farmers will regularly use biostimulant products in the near future. However, more research is needed on biostimulant properties, concentrations, and combinations for hydroponically grown plants.

## Data Availability

All data generated or analyzed during this study are included in this published article.
